# Blood-based biomarker discovery in motor neuron disease using nucleic acid-linked immuno-sandwich assay

**DOI:** 10.1093/braincomms/fcag180

**Published:** 2026-05-26

**Authors:** Hatice Bozkurt, Katy R Reid, Judith Newton, Jessica Gill, Isaac Chau, Chloe Parker, Hatice Kurucu King, Johnny Tam, Dominic Ng, Maria Stavrou, Paul Baxter, Orla Marland, Karen Burr, Amanda Heslegrave, Elena Veleva, Owen J Swann, Henrik Zetterberg, David P J Hunt, Bhuvaneish Thangaraj Selvaraj, Siddharthan Chandran, Suvankar Pal

**Affiliations:** Anne Rowling Regenerative Neurology Clinic, University of Edinburgh, Edinburgh, EH16 4SB, UK; Euan MacDonald Centre for Motor Neuron Disease Research, University of Edinburgh, Edinburgh, EH16 4SB, UK; Institute for Neuroscience and Cardiovascular Research, University of Edinburgh, Edinburgh, EH16 4TJ, UK; UK Dementia Research Institute, University of Edinburgh, Edinburgh, EH16 4SB, UK; Anne Rowling Regenerative Neurology Clinic, University of Edinburgh, Edinburgh, EH16 4SB, UK; Euan MacDonald Centre for Motor Neuron Disease Research, University of Edinburgh, Edinburgh, EH16 4SB, UK; Institute for Neuroscience and Cardiovascular Research, University of Edinburgh, Edinburgh, EH16 4TJ, UK; UK Dementia Research Institute, University of Edinburgh, Edinburgh, EH16 4SB, UK; Anne Rowling Regenerative Neurology Clinic, University of Edinburgh, Edinburgh, EH16 4SB, UK; Euan MacDonald Centre for Motor Neuron Disease Research, University of Edinburgh, Edinburgh, EH16 4SB, UK; Institute for Neuroscience and Cardiovascular Research, University of Edinburgh, Edinburgh, EH16 4TJ, UK; Anne Rowling Regenerative Neurology Clinic, University of Edinburgh, Edinburgh, EH16 4SB, UK; Euan MacDonald Centre for Motor Neuron Disease Research, University of Edinburgh, Edinburgh, EH16 4SB, UK; Institute for Neuroscience and Cardiovascular Research, University of Edinburgh, Edinburgh, EH16 4TJ, UK; Anne Rowling Regenerative Neurology Clinic, University of Edinburgh, Edinburgh, EH16 4SB, UK; Euan MacDonald Centre for Motor Neuron Disease Research, University of Edinburgh, Edinburgh, EH16 4SB, UK; Institute for Neuroscience and Cardiovascular Research, University of Edinburgh, Edinburgh, EH16 4TJ, UK; Anne Rowling Regenerative Neurology Clinic, University of Edinburgh, Edinburgh, EH16 4SB, UK; Euan MacDonald Centre for Motor Neuron Disease Research, University of Edinburgh, Edinburgh, EH16 4SB, UK; Institute for Neuroscience and Cardiovascular Research, University of Edinburgh, Edinburgh, EH16 4TJ, UK; Anne Rowling Regenerative Neurology Clinic, University of Edinburgh, Edinburgh, EH16 4SB, UK; Euan MacDonald Centre for Motor Neuron Disease Research, University of Edinburgh, Edinburgh, EH16 4SB, UK; Institute for Neuroscience and Cardiovascular Research, University of Edinburgh, Edinburgh, EH16 4TJ, UK; Anne Rowling Regenerative Neurology Clinic, University of Edinburgh, Edinburgh, EH16 4SB, UK; Euan MacDonald Centre for Motor Neuron Disease Research, University of Edinburgh, Edinburgh, EH16 4SB, UK; Institute for Neuroscience and Cardiovascular Research, University of Edinburgh, Edinburgh, EH16 4TJ, UK; UK Dementia Research Institute, University of Edinburgh, Edinburgh, EH16 4SB, UK; Anne Rowling Regenerative Neurology Clinic, University of Edinburgh, Edinburgh, EH16 4SB, UK; Euan MacDonald Centre for Motor Neuron Disease Research, University of Edinburgh, Edinburgh, EH16 4SB, UK; Institute for Neuroscience and Cardiovascular Research, University of Edinburgh, Edinburgh, EH16 4TJ, UK; UK Dementia Research Institute, University of Edinburgh, Edinburgh, EH16 4SB, UK; Anne Rowling Regenerative Neurology Clinic, University of Edinburgh, Edinburgh, EH16 4SB, UK; Euan MacDonald Centre for Motor Neuron Disease Research, University of Edinburgh, Edinburgh, EH16 4SB, UK; Institute for Neuroscience and Cardiovascular Research, University of Edinburgh, Edinburgh, EH16 4TJ, UK; UK Dementia Research Institute, University of Edinburgh, Edinburgh, EH16 4SB, UK; Anne Rowling Regenerative Neurology Clinic, University of Edinburgh, Edinburgh, EH16 4SB, UK; Euan MacDonald Centre for Motor Neuron Disease Research, University of Edinburgh, Edinburgh, EH16 4SB, UK; Institute for Neuroscience and Cardiovascular Research, University of Edinburgh, Edinburgh, EH16 4TJ, UK; UK Dementia Research Institute, University of Edinburgh, Edinburgh, EH16 4SB, UK; Anne Rowling Regenerative Neurology Clinic, University of Edinburgh, Edinburgh, EH16 4SB, UK; Euan MacDonald Centre for Motor Neuron Disease Research, University of Edinburgh, Edinburgh, EH16 4SB, UK; Institute for Neuroscience and Cardiovascular Research, University of Edinburgh, Edinburgh, EH16 4TJ, UK; UK Dementia Research Institute, University of Edinburgh, Edinburgh, EH16 4SB, UK; Anne Rowling Regenerative Neurology Clinic, University of Edinburgh, Edinburgh, EH16 4SB, UK; Euan MacDonald Centre for Motor Neuron Disease Research, University of Edinburgh, Edinburgh, EH16 4SB, UK; Institute for Neuroscience and Cardiovascular Research, University of Edinburgh, Edinburgh, EH16 4TJ, UK; UK Dementia Research Institute, University of Edinburgh, Edinburgh, EH16 4SB, UK; Department of Neurodegenerative Disease, UCL Institute of Neurology, London, WC1N 3BG, UK; UK Dementia Research Institute, University College London, London, WC1E 6BT, UK; Department of Neurodegenerative Disease, UCL Institute of Neurology, London, WC1N 3BG, UK; UK Dementia Research Institute, University College London, London, WC1E 6BT, UK; Department of Neurodegenerative Disease, UCL Institute of Neurology, London, WC1N 3BG, UK; UK Dementia Research Institute, University College London, London, WC1E 6BT, UK; UK Dementia Research Institute, University College London, London, WC1E 6BT, UK; Hong Kong Center for Neurodegenerative Diseases, Hong Kong, China; Wisconsin Alzheimer's Disease Research Center, University of Wisconsin-Madison School of Medicine and Public Health, Madison, WI, 53792, USA; Department of Psychiatry and Neurochemistry, Institute of Neuroscience and Physiology, the Sahlgrenska Academy at the University of Gothenburg, Mölndal, SE-413 45, Sweden; Anne Rowling Regenerative Neurology Clinic, University of Edinburgh, Edinburgh, EH16 4SB, UK; Euan MacDonald Centre for Motor Neuron Disease Research, University of Edinburgh, Edinburgh, EH16 4SB, UK; Institute for Neuroscience and Cardiovascular Research, University of Edinburgh, Edinburgh, EH16 4TJ, UK; UK Dementia Research Institute, University of Edinburgh, Edinburgh, EH16 4SB, UK; Anne Rowling Regenerative Neurology Clinic, University of Edinburgh, Edinburgh, EH16 4SB, UK; Euan MacDonald Centre for Motor Neuron Disease Research, University of Edinburgh, Edinburgh, EH16 4SB, UK; Institute for Neuroscience and Cardiovascular Research, University of Edinburgh, Edinburgh, EH16 4TJ, UK; UK Dementia Research Institute, University of Edinburgh, Edinburgh, EH16 4SB, UK; Anne Rowling Regenerative Neurology Clinic, University of Edinburgh, Edinburgh, EH16 4SB, UK; Euan MacDonald Centre for Motor Neuron Disease Research, University of Edinburgh, Edinburgh, EH16 4SB, UK; Institute for Neuroscience and Cardiovascular Research, University of Edinburgh, Edinburgh, EH16 4TJ, UK; UK Dementia Research Institute, University of Edinburgh, Edinburgh, EH16 4SB, UK; Anne Rowling Regenerative Neurology Clinic, University of Edinburgh, Edinburgh, EH16 4SB, UK; Euan MacDonald Centre for Motor Neuron Disease Research, University of Edinburgh, Edinburgh, EH16 4SB, UK; Institute for Neuroscience and Cardiovascular Research, University of Edinburgh, Edinburgh, EH16 4TJ, UK; UK Dementia Research Institute, University of Edinburgh, Edinburgh, EH16 4SB, UK

**Keywords:** motor neuron disease, amyotrophic lateral sclerosis, neurodegeneration, biomarker, neurofilament

## Abstract

Motor neuron disease (MND) presents with phenotypic heterogeneity, is diagnostically challenging, and has poor prognosis. The absence of accessible blood-based biomarkers has hampered progress towards precision medicine. Highly sensitive immunoassays offer considerable promise for identifying blood-based biomarkers informing underlying pathophysiology and enabling accurate diagnosis and monitoring. We report findings on parallel use of the ultra-sensitive multiplexed NUcleic Acid-Linked Immuno-Sandwich Assay (NULISA) and single molecule array (Simoa), to interrogate serum from people with MND. Sera (48 MND, 38 controls) were analysed using a NULISAseq targeted neurodegenerative panel and a Simoa neurofilament light chain (NfL) and glial fibrillary acid protein (GFAP) duplex assay. Neurofilament light and heavy chain, total tau (t-tau), phosphorylated tau (pTau)—181, pTau-217, pTau-231, fatty acid binding protein 3, amyloid beta (Aβ) 38 and Aβ40 levels were significantly elevated in MND (*P* < 0.05). Simoa and NULISAseq assays demonstrated strong correlations for NfL and GFAP (*r* > 0.90). Use of the multiplexed NULISAseq panel confirmed a well-established NfL elevation in MND, and replicated findings for other proteins from recent studies. Results add confidence in the validity and reproducibility of biomarkers identified using NULISAseq, while offering insights into the underlying pathophysiology and heterogeneity of MND.

## Teaser

Bozkurt *et al*. report the use of a novel and ultra-sensitive nucleic acid-linked immune-sandwich assay platform with a single molecular array to identify candidate biomarkers in sera of individuals with motor neuron disease compared with controls, validating and reproducing results from recent studies and providing insights into the underlying pathophysiological processes and opportunities for diagnosis and monitoring of the disease.

## Graphical Abstract

**Graphical Abstract:**
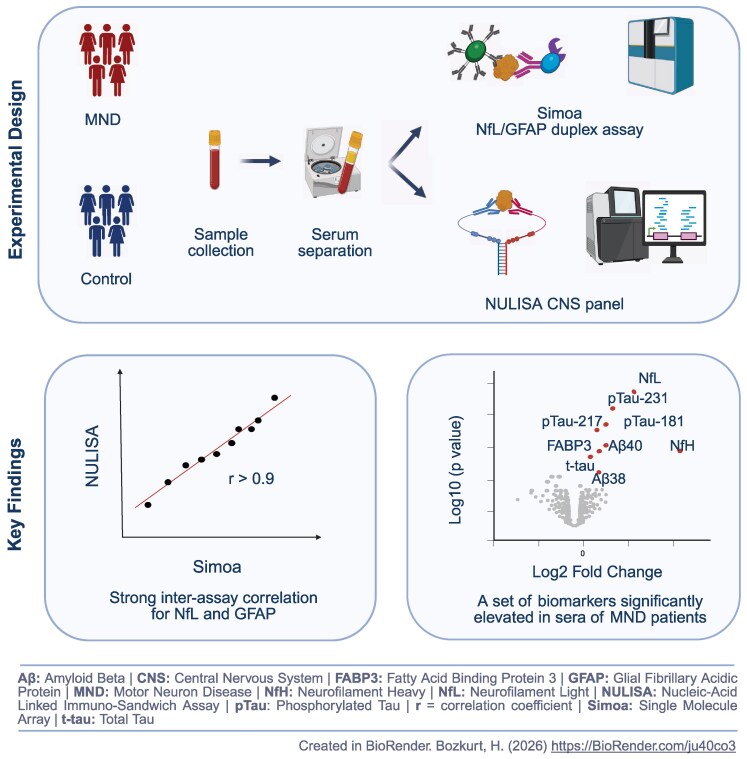

See V. Thomas and C. Pant (https://doi.org/10.1093/braincomms/fcag231) for a scientific commentary on this article.

## Introduction

Motor neuron disease (MND) is clinically heterogeneous, and diagnosis is currently based on consensus criteria and neurophysiological evaluation. Median diagnostic delay is up to 1 year from first symptom onset.^1^ The absence of validated blood-based tools to support rapid and accurate diagnosis, subtype classification, and monitoring of disease progression has limited progress in our understanding and treatment of MND. However, there is a growing interest in the utility of a range of proteins, including neurofilament light chain (NfL), neurofilament heavy chain (NfH), glial fibrillary acid protein (GFAP), phosphorylated tau (pTau), total tau (t-tau) and interferons, in supporting diagnosis, disease monitoring, and decision making for clinical trials in neurodegenerative diseases, including MND.^2-9^

The emergence of highly sensitive immunoassays offers new approaches to unlock the blood proteome to improve understanding of disease biology, diagnosis, and disease status.^10^ NUcleic acid-Linked Immuno-Sandwich Assay (NULISA) is a highly sensitive multiplexed immunoassay with unprecedented attomolar sensitivity, offering a great opportunity for biomarker discovery and validation in MND clinical studies.^10^ Here, we hypothesized that: (i) The NULISAseq multiplex assay would reliably detect NfL and GFAP, as established biomarkers of MND, and strongly correlate with results of Simoa. (ii) The highly sensitive NULISAseq multiplex assay would identify a more extended proteomic profile differentiating MND and controls, confirming findings from recent studies and adding confidence that these biomarkers may have potential for diagnosis and disease monitoring. (iii) Exploratory findings would generate further hypotheses, guiding future larger scale studies. To address our hypotheses, we used the 120-plex NULISAseq CNS Disease Panel to interrogate the proteomic profile of serum samples derived from individuals with MND linked to the Scottish MND Register (CARE-MND; Clinical Audit Research and Evaluation of Motor Neuron Disease)^11^ to identify potential markers of disease biology in a cross-sectional pilot study. We also compared NfL and GFAP results from the NULISAseq to the well-established Simoa assay.

## Materials and methods

### Patient and control samples

Serum samples from individuals with MND, and neurologically healthy controls, were collected after informed consent in Scotland, United Kingdom, between 2023 and 2024 [Scottish Regenerative Neurology Tissue Bank (SRNTB)/Lothian NRS Bioresource (REC reference: 20/ES/0061, IRAS project: ID 281531)].

### Clinical phenotyping

Clinical phenotypic data was acquired following interrogation of the Scottish Motor Neuron Disease Register digital database (CARE-MND) (REC reference: 16/SS/0156, IRAS project ID: 200777). Information was extracted regarding age at sampling, sex, MND subtype, site of onset, duration of disease, and disease severity measured using the Amyotrophic Lateral Sclerosis Functional Rating Scale Revised (ALSFRS-R).

### Sample collection and storage

A standard venepuncture technique was used for sample collection. Blood was collected in 8.5 ml serum separator tube (SST) tubes, gently inverted three to five times, and left to stand upright for 30–60 min at room temperature (RT). Samples were centrifuged at 2000×g for 10 min at RT. Serum was aliquoted into 0.5 ml Eppendorf tubes and stored at −80°C until analysis.

### NULISAseq profiling

Serum samples were analysed using the commercially available NULISAseq CNS Disease Panel 120 kit (Alamar Biosciences, Inc, Fremont, CA, USA), following the manufacturer’s instructions. Serum samples were thawed on wet ice, vortexed for 5–10 s, and centrifuged at 10 000×g for 5 min before aliquoting into the plate (30 µl), following a randomized plate map. The plate was shipped on dry ice from the University of Edinburgh to the UK Dementia Research Institute (UK DRI) Fluid Biomarker Laboratory and Biomarker Factory (UCL, London). The plate was thawed, vortexed, spun down at 1000×g for 1 min, transferred into the NULISAseq sample plate (provided in kit), and analysed on an Alamar ARGO HT System. The barcoded amplicon sequences for each target were quantified by next generation sequencing (NGS). Sample internal control (IC) median was defined as percentage deviation from the overall plate median IC reads. IC median was considered acceptable if it fell within the range of −40–40%. Detectability of a target was defined as percentage of samples that have a target signal above the lower limit of detection (LOD). A target was considered detectable if more than 20% of samples have a signal above LOD. For details on NULISAseq profiling and quality control check, see Supplementary Data. Comprehensive information for each target, including the original target names from the NULISAseq CNS Disease Panel, corresponding UniProt IDs, and quality control check, is detailed in the Supplementary Dataset.

### Simoa ultrasensitive digital enzyme-linked immunosorbent assay (ELISA)

Serum NfL and GFAP concentrations were determined using the commercially available Simoa Neurology 2- Plex B Advantage kit (Quanterix Corp., Billerica, MA, USA, item 103520) and a Simoa HD-X Analyzer (Quanterix Corp.). Analysis was performed according to the manufacturer’s instructions. Briefly, samples were thawed on wet ice, vortexed for 5–10 s, and centrifuged at 10 000×g for 5 min before aliquoting into the plate (90 µl). Samples and ICs were diluted 4× on board and measured in duplicate, while calibrators were measured neat in triplet. The results are expressed in picogram/millilitre (pg/mL). The LOD for NfL is 0.080 pg/ml and the LOD for GFAP is 0.111 pg/ml. A coefficient of variation (CV) of <20% was considered acceptable.

### Statistical analysis

Statistical analyses and data visualization were performed using Python 3.12.4, R Statistical software (v4.4.2), GraphPad Prism (10.2.3), and BioRender. Log_2_-transformed absolute concentrations of Simoa NfL and GFAP measurements and NULISA Protein Quantification (NPQ) for NULISAseq were used for analysis. Correlation assessments were performed using Spearman rank test, with results reported as correlation coefficient (*r*), associated *P*-value, and 95% confidence intervals (CIs). A volcano plot was used to visualize biomarkers that were significantly altered between disease and control groups using an independent *t*-test. To account for multiple hypothesis testing, the false discovery rate (FDR) correction was applied using the Benjamini–Hochberg procedure with a 5% threshold. Boxplots were used to present the data in the MND and control groups for a given marker, with a dashed horizontal line indicating the LOD to visualize samples above this threshold. Heatmaps were used to display the NPQ values of each biomarker as *Z*-scores relative to the mean of entire cohort. The *χ*^2^ test was used for categorical group comparison. For sensitivity and specificity analysis, a receiver operating characteristics (ROC) analysis was conducted using GraphPad Prism (10.2.3), with the area under the curve (AUC) reported alongside 95% CI. All statistical tests were two-tailed with an alpha significance level of 0.05.

## Results

**Figure 1:**
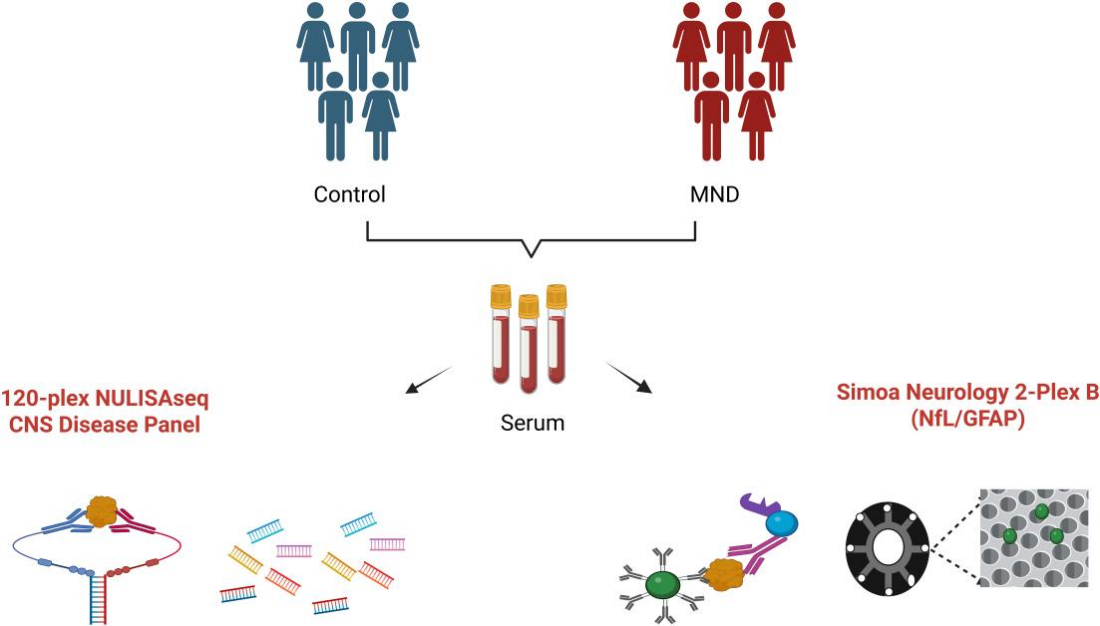
**Illustration displays the parallel experiment conducted using the NULISAseq and Simoa platforms, emphasizing key stages such as protein capture, immunocomplex formation, and data analysis for high-sensitivity biomarker detection.** Serum samples of individuals with MND and controls were analysed using a NULISAseq CNS Disease Panel 120 kit and Simoa Neurology 2-Plex b (NfL/GFAP) in parallel. In NULISAseq platform, proteins were captured by target-specific paired antibodies conjugated with DNA oligonucleotides, generating target-specific DNA barcodes for NGS. In the Simoa platform, immunocomplexes were formed with magnetic beads coated with capture antibodies and biotinylated detection antibodies. Samples were loaded into micro-wells of a single molecule array where target proteins are quantified through generation of fluorescent signals. Created in BioRender. Bozkurt, H. (2025) https://BioRender.com/9bh6uhz.

Serum samples from 48 individuals with MND and 38 controls were studied (Fig. 1). Demographic and clinical characteristics of the study population are summarized in Table 1. Characteristics of the MND cohort were generalizable to the global MND population with a median age of 66.2 years [interquartile range (IQR) 60.4–73.6], 68.8% male, 89.6% amyotrophic lateral sclerosis (ALS) subtype, 79.2% limb onset, median disease duration at the time of blood sampling 12.8 months (IQR 3.7–36.0) and median ALSFRS-R 35 (IQR 29–40). Sex distribution was not statistically different between groups for all participants (*χ*^2^, *P* = 0.06).

**Table 1:** Characteristics of individuals with MND and controls

	MND	Control
Participant—number	48	38
Sex (%)		
Female	15 (31.3)	20 (52.6)
Male	33 (68.8)	17 (44.5)
Age range at sampling—number		
25–50 years	6	3
Over 50 years	42	34
Median age at sampling—years (IQR)	66.2 (60.4–73.6)	
Region of onset—number (%)		
Limb	38 (79.2)	
Bulbar	8 (16.7)	
Respiratory	2 (4.2)	
MND subtype—number (%)		
ALS	43 (89.6)	
PLS	4 (8.3)	
Unspecified	1 (2.1)	
Median disease duration from diagnosis (IQR)	12.8 (3.7–36.0)	
Median ALSFRS-R score (IQR)	35 (29–40)	

Demographics were not available for one control. Age of controls were available as a range. ALSFRS-R assessments were conducted within 6 weeks of sampling. ALSFRS-R scores were not available for three individuals. Disease duration was determined as the number of months between diagnosis and sample collection. MND, motor neuron disease; IQR, interquartile range; ALS, amyotrophic lateral sclerosis; PLS, primary lateral sclerosis; ALSFRS-R, Amyotrophic Lateral Sclerosis Functional Rating Scale-Revised.

Two samples (one MND and one control) were excluded from statistical analysis as their IC median fell outside the cut-off range (MND: *n* = 1, IC median = 59.9%; control: *n* = 1, IC median = 50.2%) (Supplementary Dataset). Findings from 47 individuals with MND and 37 controls were included in the final statistical analysis. One additional sample was excluded from the GFAP analysis due to its CV being out of range (MND: *n* = 1, CV = 24%), resulting in a total size of 83 samples included in the final analysis (46 MND and 37 control) (Supplementary Dataset). These exclusions affected the sex distribution difference between groups for the final analysis (*χ*^2^, *P* = 0.034).

NULISAseq CNS Disease Panel analysis identified 119 proteins with detectability above 20%, as more than 20% of the samples displayed signals above the LOD. One hundred and five markers had detectability above 80%. Three proteins had detectability below 20%; therefore, were excluded in group comparison analyses: pleiotrophin, guanosine diphosphate dissociation inhibitor 1, beta-synuclein (Supplementary Fig. 1).

**Figure 2:**
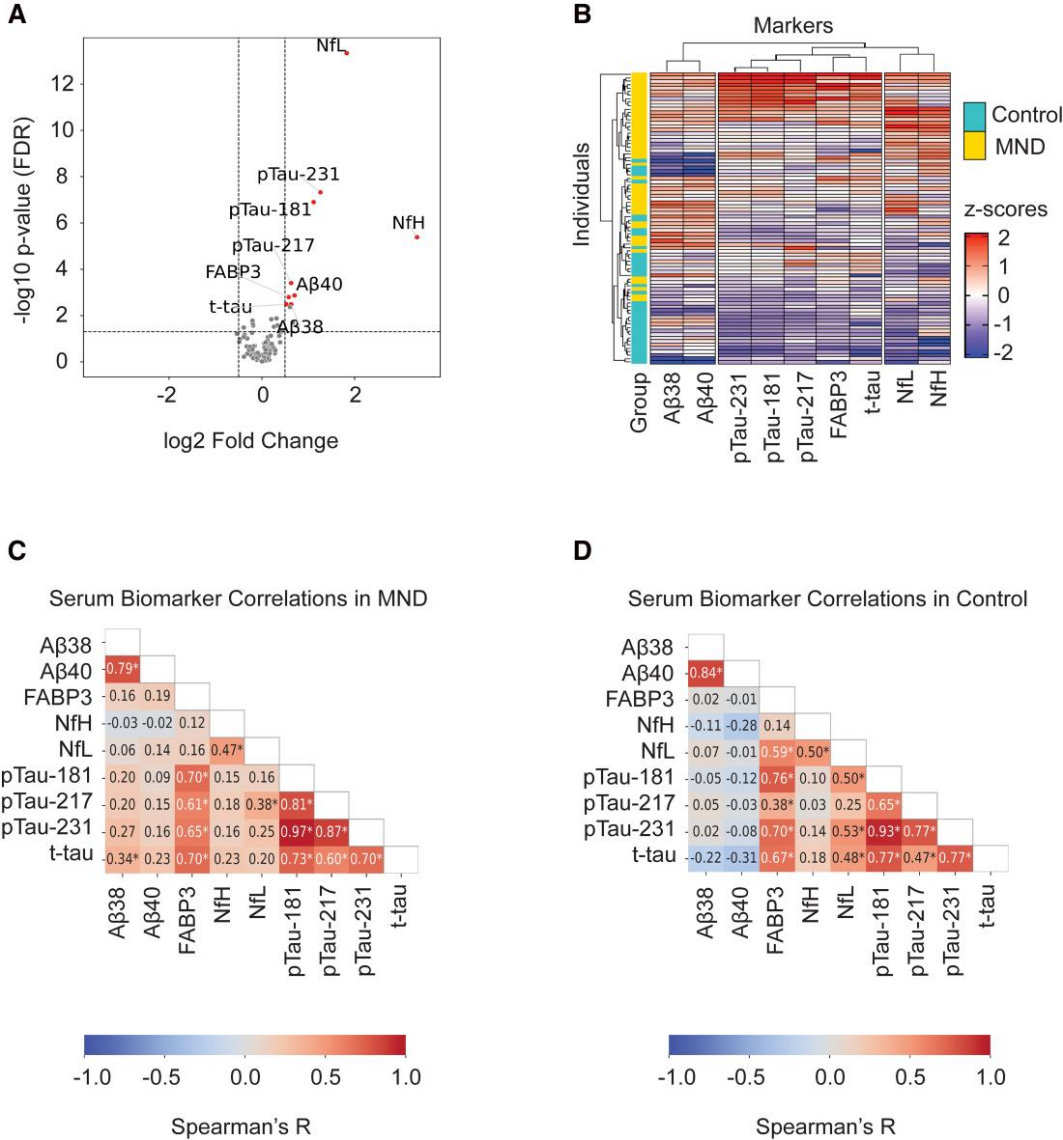
**Serum proteins show significant differences between individuals with MND and controls.** Serum samples of 86 individuals (MND, *n* = 48; control, *n* = 38) were analysed using a NULISAseq CNS Disease Panel 120 kit and Simoa Neurology 2-Plex b (NfL/GFAP) in parallel. Two samples were excluded (one MND, one control) due to their IC median values falling outside the cut-off range (>40%). This left 47 MND and 37 control samples for statistical analysis (total *n* = 84). (**A**) A volcano plot of proteins that were significantly altered between individuals with MND and controls. The horizontal dashed line represents the unadjusted *P*-value threshold of 0.05. The vertical dashed lines represent log_2_ fold changes (log_2_FC) at −0.5 and 0.5. Statistical significance was assessed using a two-tailed *t*-test, and *P*-values were adjusted for multiple testing using the Benjamini–Hochberg method. Nine proteins, that are significantly higher in MND compared with controls following the FDR correction, are as follows: NfL, log_2_FC = 1.83, *P*_adj_ < 0.001; NfH, log_2_FC = 3.34, *P*_adj_ < 0.001; pTau-231, log_2_FC = 1.26, *P*_adj_ < 0.001; pTau-181, log_2_FC = 1.12, *P*_adj_ < 0.001; pTau-217, log_2_FC = 0.63, *P*_adj_ = 0.01; t-tau, log_2_FC = 0.53, *P*_adj_ = 0.04; FABP3, log_2_FC = 0.58, *P*_adj_ = 0.03; Aβ40, log_2_FC = 0.70, *P*_adj_ = 0.03; Aβ38, log_2_FC = 0.63, *P*_adj_ = 0.04. (**B**) Heatmap based on *Z*-score hierarchical clustering shows a separation between MND and controls for the nine serum proteins. Each row represents a participant, and each column shows the protein value for that individual as a *Z*-score, with the colour indicating deviations from the mean of entire cohort. (**C**) Correlation matrix of nine significantly altered proteins (FDR corrected) in serum samples of individuals with MND and (**D**) control based on two-tailed Spearman correlations. Significant correlations were annotated with ‘*’, referring *P*-value to be <0.05.

NULISAseq analysis identified 20 differentially changed proteins in serum from individuals with MND compared with controls, of which nine remained significantly elevated following 5% FDR correction: NfL, NfH, pTau-181, pTau-217, pTau-231, t-tau, FABP3 (fatty acid-binding protein 3), Aβ38 (amyloid-beta 38), Aβ40 (amyloid-beta 40) (Fig. 2A) (*post hoc* analysis, Supplementary Fig. 4). Whilst pTau-217 was significantly elevated in MND, detectability was 8.1% in controls (3 samples out of 37 had signals above the LOD) and 31.9% in MND (15 out of 47 had signals above the LOD) (Supplementary Fig. 2).

Hierarchical clustering demonstrated a clear separation between MND and control cohorts for these nine proteins (Fig. 2B). Heatmap analysis of the correlation coefficients of every significant biomarker showed a strong positive correlation between FABP3 and pTau levels in both MND (Fig. 2C) and control samples (Fig. 2D).

An additional 11 proteins were significantly differentially regulated when FDR was not corrected, of which 7 were upregulated: amyloid-beta 42, secreted modular calcium-binding protein 1, neuropeptide Y, periostin (encoded by *POSTN*), mesothelin (encoded by *MSLN*), CCL11 (C-C motif chemokine 11—also known as eotaxin), REST (RE1-silencing transcription factor). On the other hand, four proteins were downregulated: AChE (acetylcholinesterase), S100-B (S100 calcium-binding protein B), IL-1 beta (interleukin-1 beta), DJ-1/PARK7 (protein DJ-1/Parkinson disease protein 7) (Supplementary Fig. 3).

**Figure 3:**
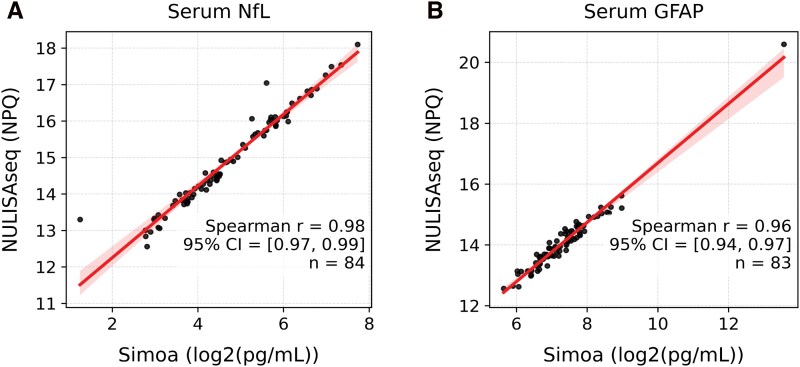
**Measurements of serum NfL and GFAP demonstrating a strong correlation between Simoa and NULISAseq assays.** Serum samples of 86 individuals (MND, *n* = 48; control, *n* = 38) were analysed using the Simoa and NULISAseq platforms in parallel. Two samples were excluded (one MND, one control) due to their IC median values falling outside the cut-off range (>40%). This left 47 MND and 37 control samples for statistical analysis of NfL correlation (total *n* = 84). One additional sample was excluded from the GFAP analysis due to its CV being out of range (>20%), resulting in a total sample size of 83 (46 MND and 37 control). Absolute quantification values of Simoa were log_2_ transformed [i.e. log_2_(pg/ml)] before conducting a correlation analysis. Each dot represents the result of a subject, with Simoa measurements [log_2_(pg/ml)] on *x*-axis and NULISAseq measurements (NPQ) on *y*-axis. Serum measurements showed a significant correlation between Simoa and NULISAseq assays for (**A**) NfL [Spearman’s rank coefficient *R* = 0.98, 95% CI = (0.97, 0.99), *P* < 0.001] and (**B**) GFAP [Spearman’s rank coefficient *R* = 0.96, 95% CI = (0.94, 0.97), *P* < 0.001].

To evaluate congruence of the NULISAseq data with an established and validated analysis platform, NfL and GFAP levels were also measured in MND and control samples using Simoa (Neurology—2 Plex B duplex assay, Quanterix). Results confirmed a significant correlation between the NULISAseq and Simoa assays for both NfL (Spearman’s rank coefficient *R* = 0.98, *P* < 0.001) (Fig. 3A) and GFAP (Spearman’s rank coefficient *R* = 0.96, *P* < 0.001) (Fig. 3B).

We also conducted an exploratory analysis investigating correlations between nine significantly differentiated biomarkers (NfL, NfH, pTau-181, pTau-217, pTau-231, t-tau, FABP3, Aβ38 and Aβ40) and clinical characteristics of individuals with MND. We found no significant correlation, except for NfL and pTau-217 demonstrating a significant inverse correlation with disease duration (Supplementary Figs 5–8). To evaluate the discriminative sensitivity and specificity performance of biomarkers, ROC analysis was conducted for nine significant biomarkers. Among them, NfL demonstrated the highest discriminatory performance between MND and controls [AUC = 0.92, 95% CI = (0.86–0.97)], followed by pTau-231 [AUC = 0.85, 95% CI = (0.77–0.93)] and pTau-181 [AUC = 0.83, 95% CI = (0.75–0.92)] (Supplementary Fig. 9). Finally, we conducted a subgroup analysis comparing the proteomic profile of individuals with ALS (the commonest subtype of MND) with controls, which replicated key findings for the total group of participants with MND namely significantly elevated levels of NfL, NfH, pTau-181, pTau-217 and pTau-231 (Supplementary Figs 10 and 11).

## Discussion

We report on use of the highly sensitive NULISAseq CNS panel to identify distinct blood protein signatures in MND compared with healthy controls using a cross-sectional design of individuals representative of the wider MND population.^12^

We have independently replicated key findings from a recently published study by Thomas *et al*.,^13^ that interrogated plasma samples of ALS patients using NULISAseq CNS panel. Our results also demonstrated substantial overlap in findings with other recent studies that used the NULISAseq CNS panel to investigate samples from individuals with ALS harbouring genetic mutations.^14,15^

Differences in this study include use of serum samples and inclusion of people with different subtypes of MND. We also conducted further assay validation to evaluate the reliability and reproducibility of the NULISAseq multiplex against the well-established Simoa assay and demonstrated a strong correlation between two assays for two commonly used biomarkers in MND research, NfL and GFAP.

Whilst there has been considerable variability in recently reported studies with the assay of serum or plasma, a recent study by Durcan *et al*.^16^ suggests that NULISAseq performs with good consistency across different sample types. The significant replication and overlap of our findings with recent reports further indicates consistent assay performance using either serum or plasma in MND, particularly for NfL, GFAP and phosphorylated tau.

Results demonstrate upregulation of multiple markers of neurodegeneration in MND compared with controls including NfL and NfH. These non-specific markers of axonal injury have been reported as indicators of progressive neurodegeneration in MND, including recent use as a biomarker in evaluation of the efficacy of tofersen in clinical trials for individuals with the *SOD1* (superoxide dismutase 1) genetic subtype of MND.^5,17^

Our data also demonstrated significant upregulation of total and pTau in MND compared with controls. Tau protein is known for its key role in microtubule assembly and stabilization and has been studied extensively in Alzheimer’s disease where it is implicated in formation of pathological neurofibrillary tangles in the brain.^18^ Despite limited previous reports in MND, recent studies have described altered tau levels in CSF and blood and postulate altered tau metabolism.^19,20^ This includes increased t-tau and pTau levels in blood and considerably low pTau levels in CSF in ALS compared with controls.^7,8,21^ Recent studies using samples from pwMND and using the NULISAseq platform also support these findings with a similar plasma tau profile in ALS.^13-15^ Results from our study build on this recent research with findings of a substantial increase in serum t-tau, pTau-181, pTau-217 and pTau-231 levels in individuals with MND compared with controls, strengthening their potential utility as biomarkers in MND. The assays used react with both brain-derived tau (short tau) and big-tau, sometimes called peripheral tau. In the CNS, big tau has been known to be mostly expressed in cell types whose processes extend to the periphery, such as dorsal root ganglion, superior posterior ganglion, and spinal motor neurons.^22,23^ These data suggest that pTau increase (measured using assays that do not differentiate brain-derived from big-tau) selectively in serum but not in CSF may relate to spinal motor neuron degeneration with biomarker release directly into the bloodstream and not the CSF. pTau may therefore be a helpful biomarker reflecting distinct pathology, and of utility for disease monitoring including in clinical trials. Further larger studies linked to clinical phenotyping may help evaluate this further. A recently published study examining serum and muscle tissue from individuals with ALS suggests that pTau-217 and pTau-181 are significantly elevated in serum samples.^24^ This study also observed sarcoplasmic reactivity to pTau-181 and pTau-217 in atrophic muscle fibres in ALS, with no reactivity found in normal or hypertrophic fibres. The study indicates a correlation between other muscle markers like troponin and pTau level, underscoring the possibility of peripheral tissue source of pTaus.

Another intriguing finding of our study was elevation of FABP3, an intracellular protein involved in lipid transport and cell energy metabolism, and its differential regulation in blood in MND.^13,15^ Recent studies have evaluated its role as a biomarker in Alzheimer’s disease, stroke and Parkinson’s disease,^25^ including contributing to α-synuclein oligomerization, indicating microglial activation, promoting ischaemic neuronal damage, and inducing endoplasmic reticulum stress.^26-29^

Interestingly, our results included a significant and strong correlation between FABP3 and all tau proteins. Given that recent evidence suggests a peripheral source of pTaus in MND, our findings raise the question of whether FABP3 could be linked to peripheral nervous system or muscle pathology, separate to a possible CNS related microglia activation. Whilst data is limited, some studies suggest FABP3 can be a potential marker linked to skeletal muscles weakness in aging and inflammatory muscle diseases.^29,30^ Correlation with paired CSF and peripheral tissues may be helpful although beyond the scope of this study.

Additionally, we noted a substantial change in amyloid proteins, a key protein in amyloid plaques in Alzheimer’s disease,^31^ in MND compared with controls. As reported in recent studies,^13,32,33^ our findings underscore the potential of amyloid proteins as supplementary biomarker for MND, warranting further studies to better understand the interaction and significance of amyloid with other neurodegenerative markers including tau and neurofilament proteins in MND. Autopsy studies may progress understanding of whether this finding represents co-pathology or is specific to neurodegeneration in MND.

Heatmap analysis demonstrated significant clustering of nine proteins NfL, NfH, t-tau, pTau-181, pTau-231, pTau-217, FABP3, Aβ38 and Aβ40 distinguishing MND and control samples. This observation suggests these proteins may serve as key indicators of disease pathophysiology and offer potential as biomarkers for disease diagnosis. Furthermore, clustering patterns may point to clinical subgroups within the MND cohort characterized by tau and amyloid pathology, providing insights into disease heterogeneity.

Recent research has suggested neuroinflammation may play an important role in the pathogenesis of MND, including increased reactivity of glial cells upregulation of pro-inflammatory markers in circulatory system.^4,9^ Results from this study identified substantial alterations in the levels of several proteins implicated in neuroinflammation, including CCL11, IL-1 beta and S100-B; however, these findings did not remain significant following FDR correction. No significant differences were observed in several other neuroinflammation-related markers, including GFAP, IL-6 (interleukin-6), tumour necrosis factor-α and other cytokines, regardless of FDR correction. However, it is important to note that due to their small effect size and our modest sample size, many of these markers did not achieve strong differences in statistical significance.

We acknowledge limitations of this study including the modest sample size, which restricted subgroup analyses related to genetic and clinical phenotypes, and differences between the age and sex of disease and control groups. There is a need for future replication studies in larger well-characterized and longitudinal cohorts, including investigations of paired CSF and blood samples, and autopsy brain and peripheral nervous system tissues as well as other peripheral tissues such as skeletal muscles.

## Conclusion

Understanding the interplay between neurodegenerative, systemic and neuroinflammatory processes in the pathophysiology of MND is crucial to understanding phenotypic heterogeneity and the selection of targeted therapies. Deployment of the NULISAseq neurodegenerative multiplex in serum samples derived from individuals with MND has identified a distinct biomarker profile, confirming upregulation of proteins associated with neuronal and astrocytic degeneration including NfL, NfH, t-tau, pTau-181, pTau-217, pTau-231, Aβ38, Aβ40 and FABP3. Results from our study provide insights into the underlying pathophysiology of MND and provide further confidence in the validity and robustness of the NULISAseq assay.

## Supplementary Material

fcag180_Supplementary_Data

## Data Availability

Raw data is available to share upon request from the authors. Links to the code repository are available via HaticeBzkrt/brain_comm_2025_R and HaticeBzkrt/brain_comms_Bozkurt-et-al_2025_python.
