# Supplementary material for: Blood-based biomarker discovery in motor neuron disease using nucleic acid-linked immuno-sandwich assay
Source: Brain Commun. 2026 May 26;8(3):fcag180. doi: 10.1093/braincomms/fcag180 (PMC13312953; doi:10.1093/braincomms/fcag180)
Supplement: fcag180_Supplementary_Data [file fcag180_supplementary_data.zip › Supplementary_Data.pdf]

SUPPLEMENTARY DATA

Immunocomplex formation in NULISAseq

Comprehensive descriptions of the assay were provided by Feng, et al. in a recent study[1]. Briefly, samples are aliquoted into the 96-PCR well and loaded into the ARGO HT Alamar NULISA platform. Target specific paired antibodies, which are conjugated with oligonucleotides, are added into the samples to capture targets and form immunocomplexes. Then, the dT coated beads are added to the sample mix to capture immunocomplexes via binding to the poly-A tails of antibodies. Following this, the sample mixtures are washed to clear the unbounded antibodies alongside sample matrix components. Following this, dT bead captured immunocomplexes are released to the low-salt buffer. To re-capture the released immunocomplexes, another set of beads coated with streptavidin are added into the samples. This was, then, followed by the second wash to further clear unbound antibodies. Finally, after the second wash, the oligo tails of the paired antibodies ligate to form a DNA barcode. This DNA barcodes are target specific which are used in the sequencing (NGS) for quantification of each target accordingly.

Data analysis and QC check in NULISAseq

NULISA kits include various kind of controls to be used in data analysis and QC check. Each well has an internal control (IC) – a spiked-in mCherry protein, which are used during the normalization of the data. Each plate includes four negative controls (NCs), which do not contain any target. The value coming from NCs well are considered as "background noise" and used in the calculation of LOD. Sample control (SC, n = 3) are pooled plasma controls samples that are used in assessing intra-assay variations.

IC median in NULISAseq

An IC median is a central value of all IC reads measured across the wells on the plate, representing the midpoint of the data distribution. ±40% threshold is set as an acceptable range for variation in a sample's IC reading.

Calculation of NPQ in NULISAseq

Next Generation Sequencing (NGS) produces reads for each target in each sample. Firstly, these reads are normalized using internal control (IC). Then, the normalized data are re-scaled by multiplication with a factor of 10^4. Finally, the data is transformed into log2 scale, making up NPQ value for each target. To avoid zero value issue during the log transformation, +1 was added to each data point just before log2 transformation.

LOD in NULISAseq

The results from negative controls (NCs) are utilized in the calculation of the Limit of Detection (LOD) for each target. Any signal observed in the negative controls is considered as "background noise" for that target. Before calculating the LOD, the counts from the NCs are normalized. Next, the mean and standard deviation (SD) of the normalized NC data is calculated. These values are, then, used in the following formula to calculate the unlogged LOD: Unlogged LOD = Mean of normalized NC counts + 3 × SD of normalized NC counts.

Next, the unlogged LOD is re-scaled (multiplied by 10^4) and transformed into a log2 scale. Since the negative controls (NCs) do not contain any target, the read counts from these controls are expected to be zero or very low. If no signal detected for a target in negative controls, meaning "zero", as the zero cannot be transformed into a log2 value, it is treated as zero directly. Following this transformation, each target is assigned a target LOD NPQ value that represents the background noise from the negative controls for that specific target. To determine the detectability of a target within a sample, the target LOD NPQ is subtracted from NPQ value of a target in a sample, resulting in the NPQ-LOD signal (NPQ minus LOD). A target is considered detectable in a sample if its NPQ-LOD value is greater than zero. Detectability % of a target is calculated as the proportion of samples within a cohort that has NPQ-LOD value above zero.

SUPPLEMENTARY FIGURES

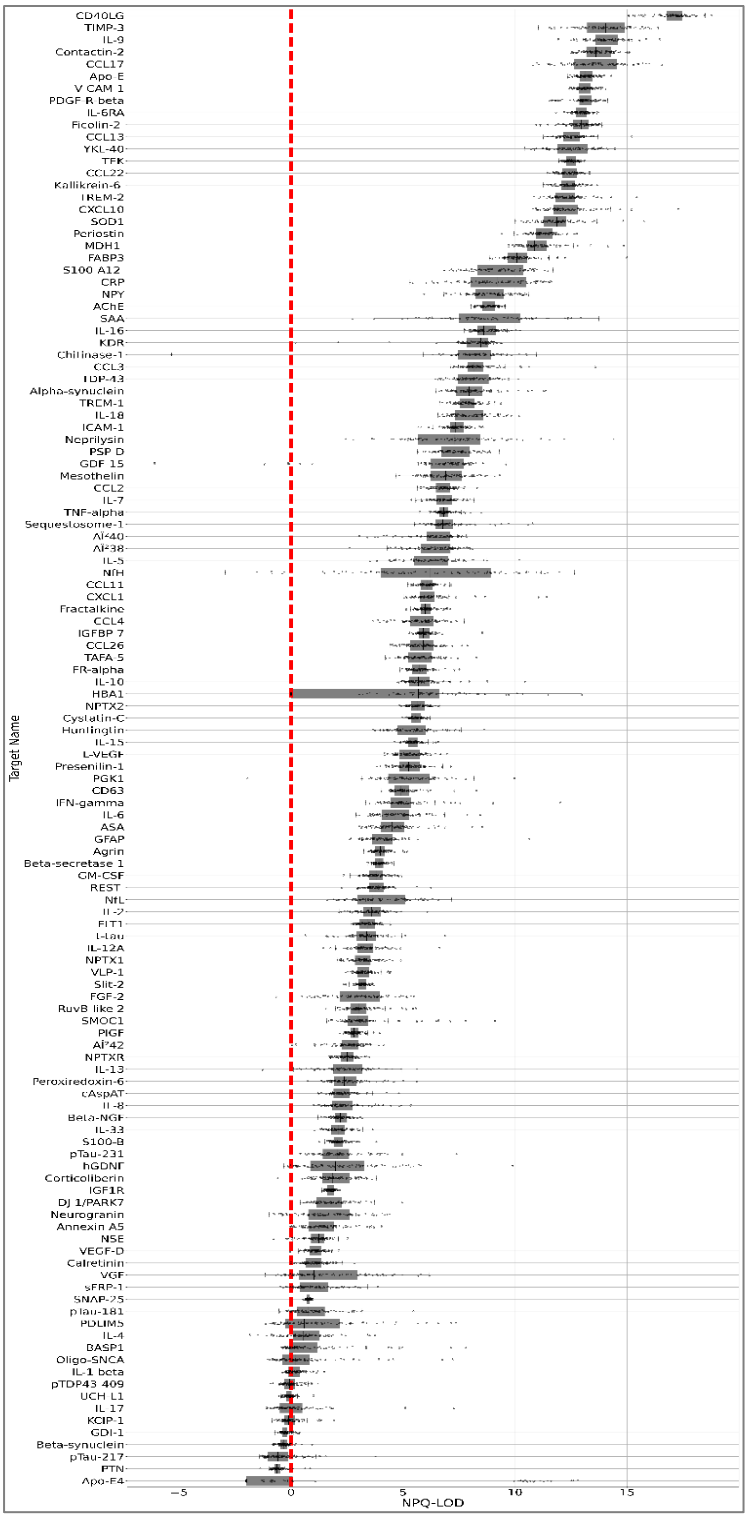

Supplementary Figure 1 – NULISAseq CNS Disease Panel shows high detectability percentage for targets

Serum samples of 86 individuals (MND, n = 48; non-ND control, n= 38) were analysed using a NULISAseq™ CNS Disease Panel 120 kit. Two samples were excluded (1 MND, 1 Control) due to their IC median values falling outside the cut-off range (> 40%). This resulted in a final sample size of 84, comprising 47 MND and 37 Control samples. A figure illustrates detectability of targets in each sample, calculated as NPQ minus LOD. Each data point represents the NPQ-LOD value for individual subjects per target, with NPQ-LOD values on the x-axis, and target names on the y-axis. A zero point serves as the reference, with NPQ-LOD values above zero indicating target detectability. The boxes represent 50% of data (IQR); horizontal middle lines inside the boxes show median value; whiskers are 1.5 times of the interquartile range (IQR) and extend from IQR; outliers are any data point outside of whiskers. Comprehensive details for each sample and target, including the original target names from the NULISAseq CNS Disease Panel, full protein names, and corresponding UniProt IDs, are available in the supplementary dataset.

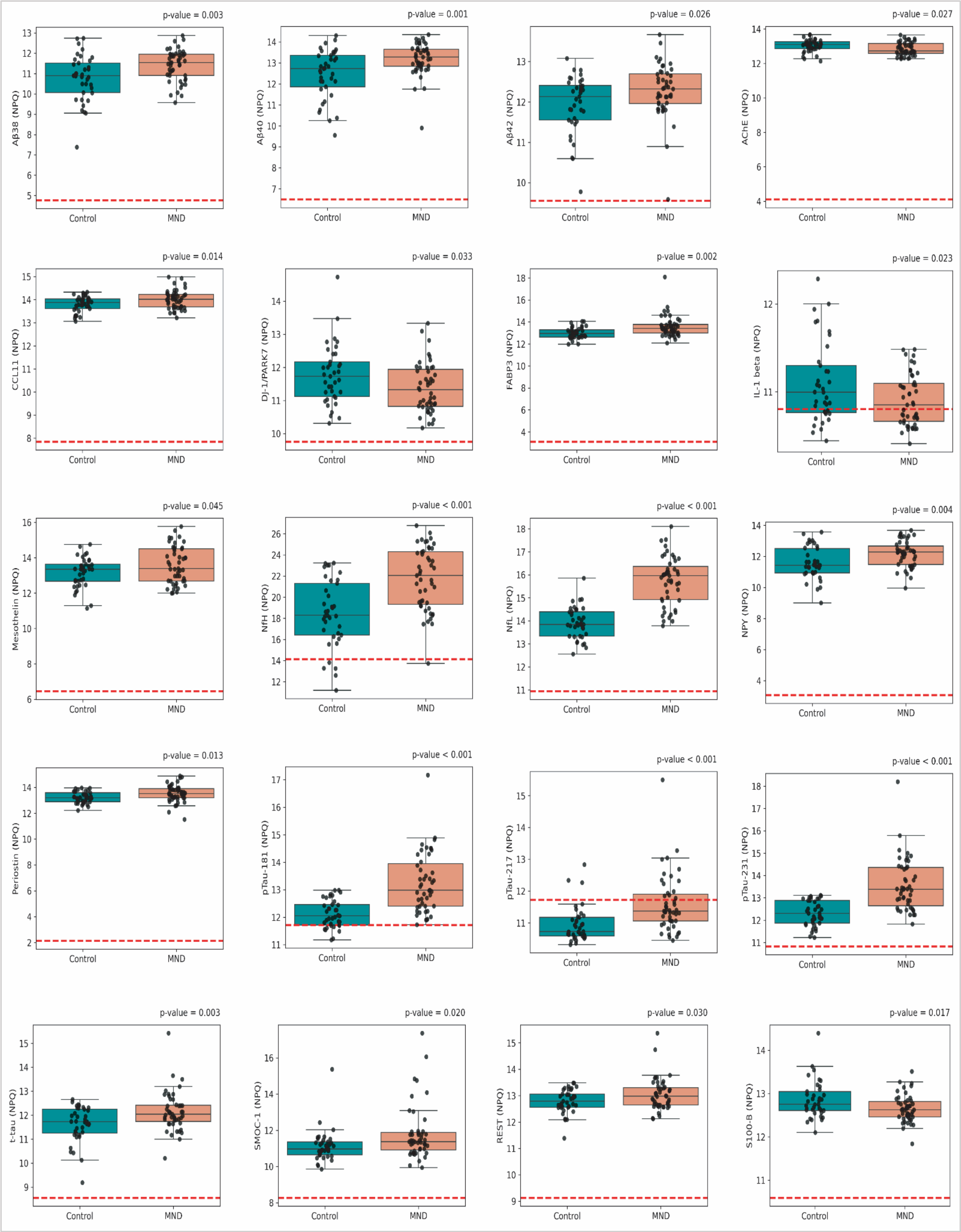

Supplementary Figure 2 – Candidate biomarkers identified using NULISAseq CNS Disease Panel display high detectability percentage

Serum samples of 86 individuals (MND, n = 48; control, n= 38) were analysed using a NULISAseq™ CNS Disease Panel 120 kit. Samples with IC median of > 40% were excluded from further analysis (MND, n= 1 & control, n = 1). This resulted in a final sample size of 84 for statistical analysis, comprising 47 MND and 37. Boxplots display data per subject in MND and control groups for each significant protein. Each dot corresponds to the result of a subject for the specified protein. The boxes represent 50% of data (IQR), horizontal middle lines inside the boxes show median value; whiskers are 1.5 times of the interquartile range (IQR) and extend from IQR; outliers are any data point outside of whiskers. The p-value shows the result of a two-tailed t-test (unadjusted). The dashed red horizontal line shows the LOD for each marker (Aβ38, LOD = 4.76 NPQ, Detectability = 100%; Aβ40, LOD = 6.50 NPQ, Detectability = 100%; Aβ42, LOD = 9.55 NPQ, Detectability = 100%; AChE, LOD = 4.11 NPQ, Detectability = 100%; CCL11, LOD = 7.85 NPQ, Detectability = 100%; DJ-1/PARK7, LOD = 9.76 NPQ, Detectability = 100%; FABP3, LOD = 3.10 NPQ, Detectability = 100%; IL-1 beta, LOD = 10.80 NPQ, Detectability = 58.33%; Mesothelin, LOD = 6.45 NPQ, Detectability = 100%; NfH, LOD = 14.13 NPQ, Detectability = 92.86%; NfL, LOD = 10.94 NPQ, Detectability = 100%; NPY, LOD = 3.07 NPQ, Detectability = 100%; Periostin, LOD = 2.13 NPQ, Detectability = 100%; pTau-181, LOD = 11.71 NPQ, Detectability = 89.29%; pTau-217, LOD = 11.72 NPQ, Detectability = 21.43%; pTau-231, LOD = 10.82 NPQ, Detectability = 100%; t-tau, LOD = 8.55 NPQ, Detectability = 100%; SMOC-1, LOD = 8.26 NPQ, Detectability = 100%; REST, LOD = 9.12 NPQ, Detectability = 100%; S100-B, LOD = 10.59 NPQ, Detectability = 100%).

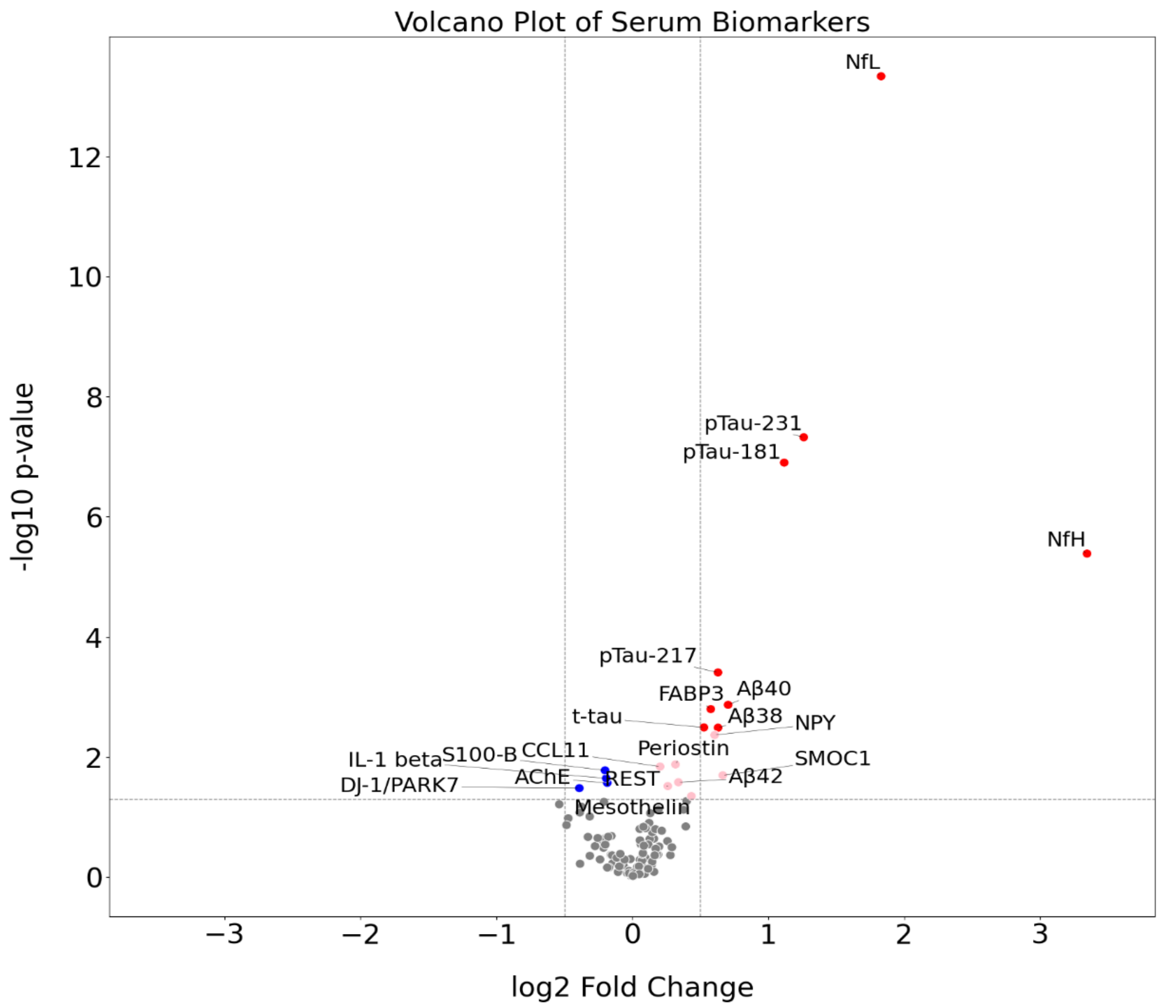

Supplementary Figure 3 – The NULISAseq CNS panel identified twenty biomarkers as substantially different between control and MND groups

Serum samples of 86 individuals (MND, n = 48; non-ND control, n= 38) were analysed using a NULISAseq™ CNS Disease Panel 120 kit. Samples with IC median of > 40% were excluded from the analysis (MND, n= 1; non-ND, n = 1). This resulted in a final sample size of 84 for statistical analysis, comprising 47 MND and 37. A volcano plot presents biomarkers that were significantly altered between individuals with MND and non-ND controls. The horizontal dashed line represents the unadjusted p value threshold of 0.05. Statistical significance was assessed using a two-tailed t-test, and p-values were adjusted for multiple testing using the Benjamini-Hochberg method. Grey coloured dots present proteins which did not show a significant difference between groups. Pink coloured dots highlight proteins that were significantly higher in MND only when p value was not corrected (Aβ42, log2FC = 0.34, P_{unadj} = 0.03; NPY, log2FC = 0.61, P_{unadj} <0.01; SMOC-1, log2FC = 0.67, P_{unadj} = 0.02; CCL11, log2FC = 0.20, P_{unadj} = 0.01; Periostin, log2FC = 0.32, P_{unadj} = 0.01; Mesothelin, log2FC = 0.43, P_{unadj} = 0.04; REST, log2FC = 0.26, P_{unadj} = 0.03). Blue coloured dots show proteins that are significantly lower in MND only when p value was not corrected (AChE, log2FC = -0.18, P_{unadj} = 0.03; S100-B, log2FC = -0.20, P_{unadj} = 0.02; IL-1 beta, log2FC = -0.19, P_{unadj} = 0.02, DJ-1/PARK7, log2FC = -0.39, P_{unadj} = 0.03). Red coloured dots represent proteins that remain significantly higher in MND after FDR correction (NfL, log2FC = 1.83, P_{adj} <0.001; NfH, log2FC = 3.34, P_{adj} <0.001; pTau-231, log2FC = 1.26, P_{adj} <0.001; pTau-

181, log2FC = 1.12, P_{adj} <0.001; pTau-217, log2FC = 0.63, P_{adj} = 0.01; t-tau, log2FC = 0.53, P_{adj} = 0.04; FABP3, log2FC = 0.58, P_{adj} = 0.03; Aβ40, log2FC = 0.70, P_{adj} = 0.03; Aβ38, log2FC = 0.63, P_{adj} = 0.04).

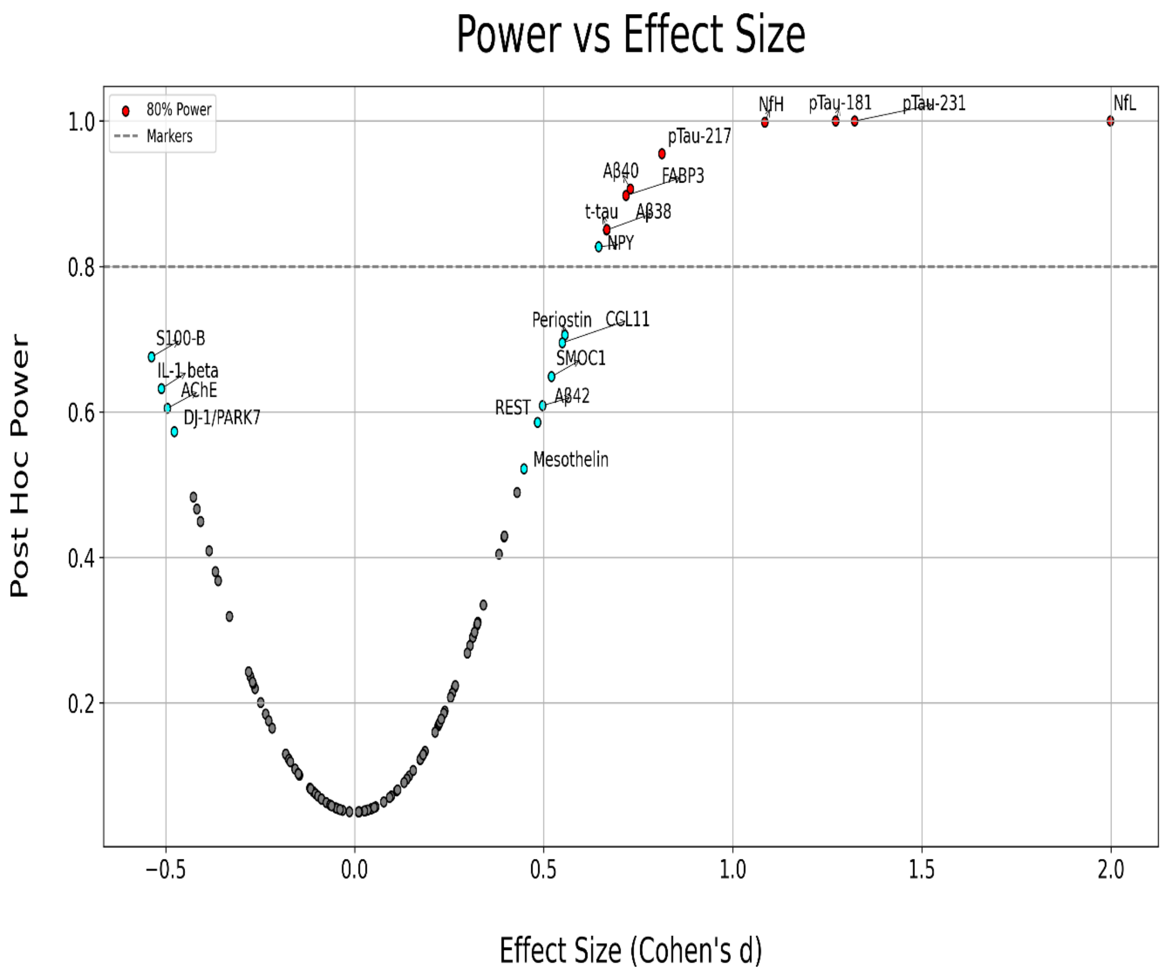

Supplementary Figure 4 – Post hoc analysis shows strong power for ten biomarkers.

Serum samples of 86 individuals (MND, n = 48; non-ND control, n= 38) were analysed using a NULISAseq™ CNS Disease Panel 120 kit. Samples with IC median of > 40% were excluded from the analysis (MND, n= 1; non-ND, n = 1). This resulted in a final sample size of 84 for statistical analysis, comprising 47 MND and 37. Each dot represents the result of a target from the NULISAseq CNS panel, with effect size on x-axis and post-hoc power on y-axis. Red coloured dots represent proteins that remain significantly higher in MND after FDR correction (NfL, Cohen’s d = 2.0, power = 1.0; pTau-231, Cohen’s d = 1.32, power = 1.0; pTau-181, Cohen’s d = 1.27, power = 1.0; NfH, Cohen’s d = 1.09, power = 1.0; pTau-217, Cohen’s d = 0.81, power = 0.95; Aβ40, Cohen’s d = 0.73, power = 0.91; FABP3, Cohen’s d = 0.72, power = 0.90; Aβ38, Cohen’s d = 0.67, power = 0.85; t-tau, Cohen’s d = 0.67, power = 0.85). Cyan coloured dots highlight proteins that were significantly different between the groups only when p value was not corrected (NPY, Cohen’s d = 0.65, power = 0.83; Periostin, Cohen’s d = 0.56, power = 0.71 ; CCL11, Cohen’s d = 0.55, power = 0.70; S100-B, Cohen’s d = - 0.54, power = 0.68; SMOC-1, Cohen’s d = 0.52, power = 0.65; IL-1 beta, Cohen’s d = -0.51, power = 0.63; AChE,

Cohen's d = -0.50, power = 0.61; Aβ42, Cohen's d = 0.50, power = 0.61; REST, Cohen's d = 0.48, power = 0.59; DJ-1/PARK7, Cohen's d = -0.48, power = 0.57; Mesothelin, Cohen's d = 0.45, power = 0.52) Grey coloured dots present proteins which did not show a significant difference between groups regardless of FDR correction.

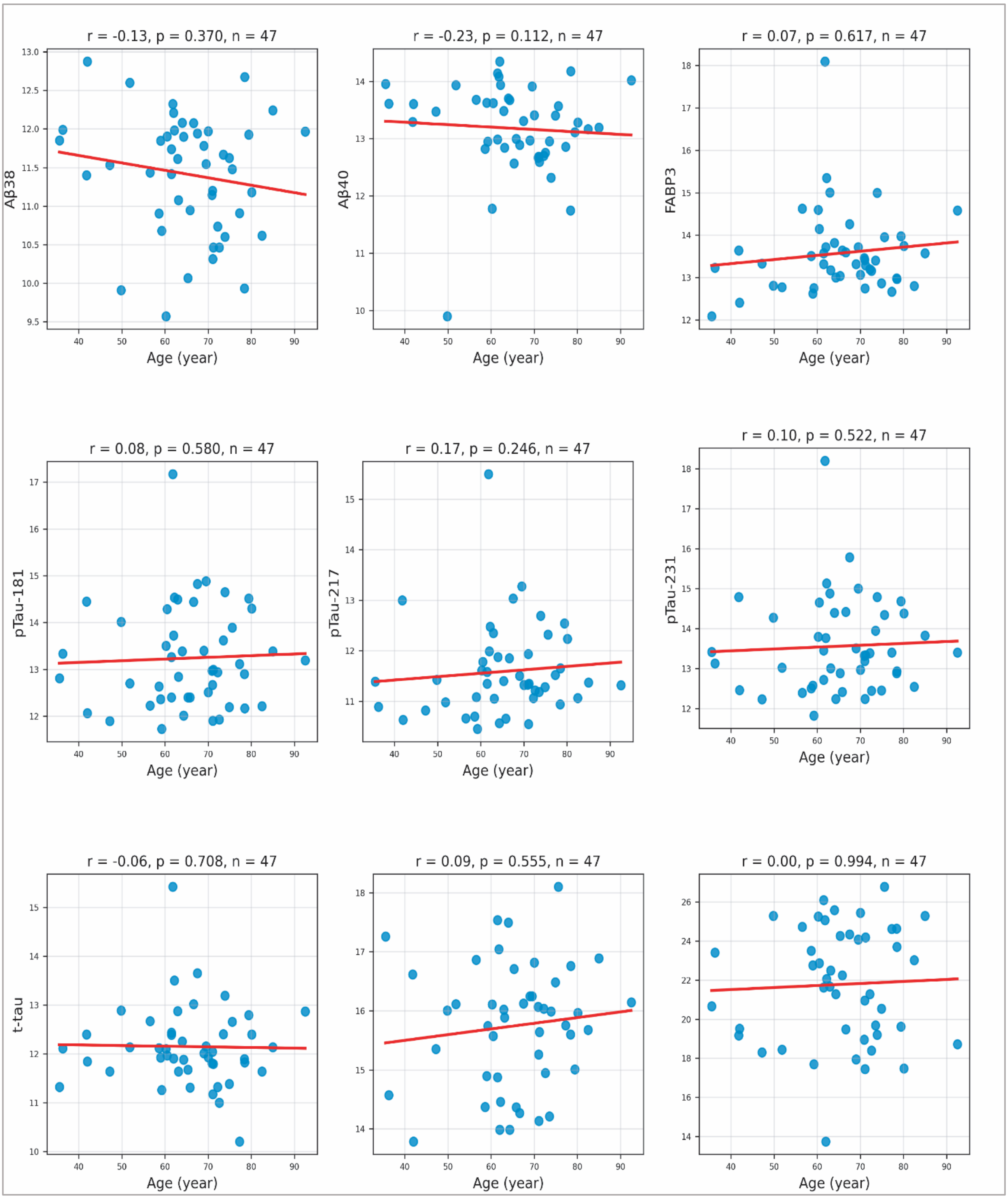

Supplementary Figure 5 – No significant correlation was observed between age and nine differentially expressed biomarkers in MND.

Serum samples from 48 MND patients were analysed using the NULISAseq™ CNS Disease Panel 120 kit. Samples with an IC median > 40% were excluded from further analysis (MND, n=1), resulting in a final sample size of 47 for statistical analysis. Each dot represents the result of a subject, with age at sampling (year) on x-axis and NULISAseq measurements (NPQ) on y-axis. Serum measurements showed no significant correlation between age and NULISAseq markers for NfL (Spearman's Rank Coefficient R = 0.09, 95% CI = [-0.20, 0.37], p = 0.555), NfH (Spearman's Rank Coefficient R = 0.00, 95% CI = [-0.29, 0.29], p = 0.994), Aβ38

(Spearman's Rank Coefficient R = -0.13, 95% CI = [-0.41, 0.16], p = 0.370), Aβ40 (Spearman's Rank Coefficient R = -0.23, 95% CI = [-0.49, 0.06], p = 0.112), FABP3 (Spearman's Rank Coefficient R = 0.07, 95% CI = [-0.22, 0.35], p = 0.617), pTau-181 (Spearman's Rank Coefficient R = 0.08, 95% CI = [-0.21, 0.36], p = 0.580), pTau-217 (Spearman's Rank Coefficient R = 0.17, 95% CI = [-0.12, 0.44], p = 0.246), pTau-231 (Spearman's Rank Coefficient R = 0.10, 95% CI = [-0.20, 0.37], p = 0.522), t-tau (Spearman's Rank Coefficient R = -0.06, 95% CI = [-0.34, 0.23], p = 0.708).

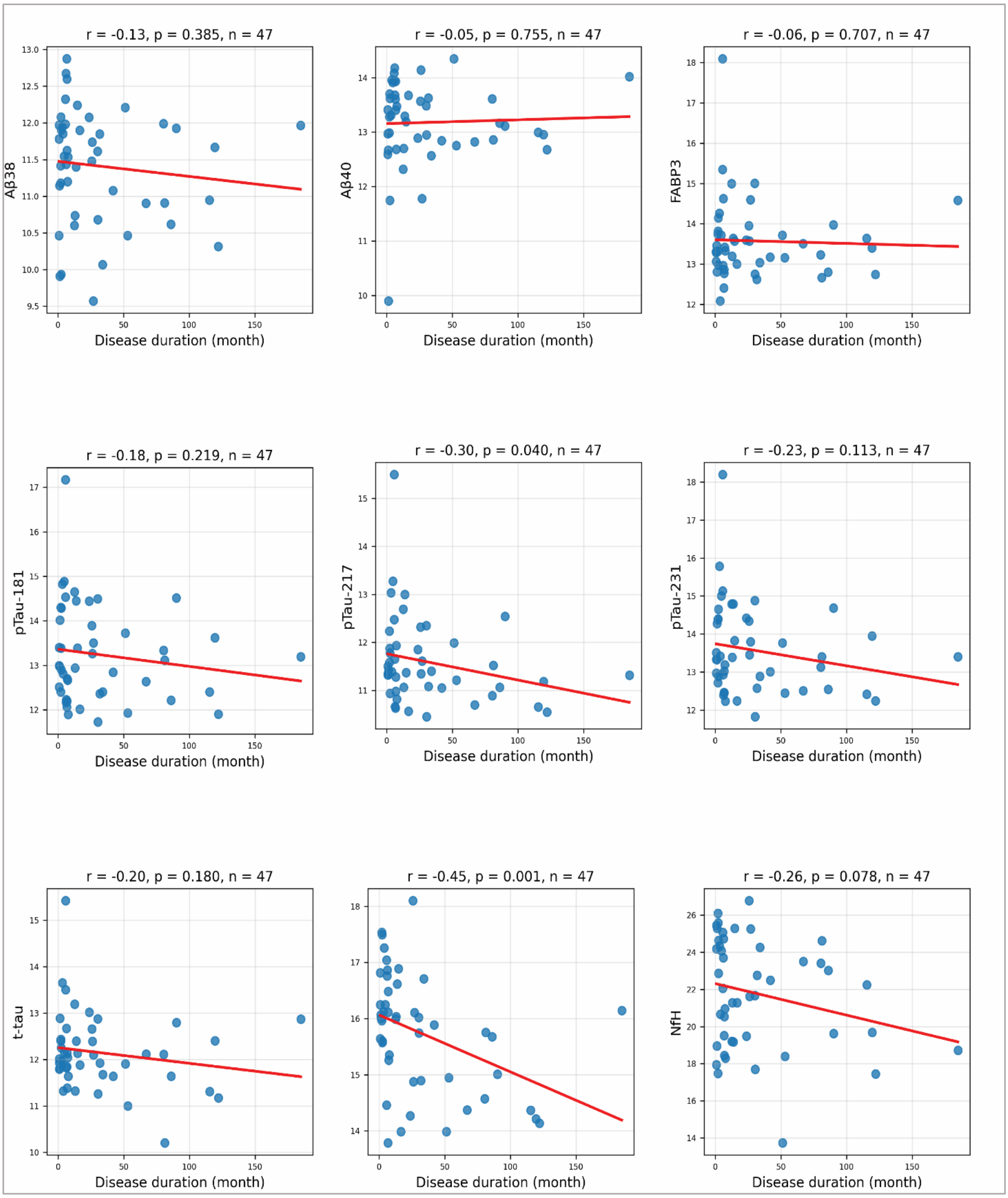

Supplementary Figure 6 – Serum NfL and pTau-217 levels showed significantly inverse correlation with disease duration from diagnosis.

Serum samples from 48 MND patients were analysed using the NULISAseq™ CNS Disease Panel 120 kit. Samples with an IC median > 40% were excluded from further analysis (MND, n=1), resulting in a final sample size of 47 for statistical analysis. Each dot represents the result of a subject, with disease duration from diagnosis (month) on x-axis and NULISAseq measurements (NPQ) on y-axis. Serum measurements showed a significant correlation between disease duration and NULISAseq markers for NfL (Spearman's Rank Coefficient R = -0.45, 95% CI = [-0.66, -0.19], p = 0.001), pTau-217 (Spearman's Rank Coefficient R = -0.30,

95% CI = [-0.54, -0.02], p = 0.040), but not for NfH (Spearman's Rank Coefficient R = -0.26, 95% CI = [-0.51, 0.03], p = 0.078), Aβ38 (Spearman's Rank Coefficient R = -0.13, 95% CI = [-0.40, 0.16], p = 0.385), Aβ40 (Spearman's Rank Coefficient R = -0.05, 95% CI = [-0.33, 0.24], p = 0.755), FABP3 (Spearman's Rank Coefficient R = -0.06, 95% CI = [-0.34, 0.23], p = 0.707), pTau-181 (Spearman's Rank Coefficient R = -0.18, 95% CI = [-0.45, 0.11], p = 0.219), pTau-231 (Spearman's Rank Coefficient R = -0.23, 95% CI = [-0.49, 0.06], p = 0.113), t-tau (Spearman's Rank Coefficient R = -0.20, 95% CI = [-0.46, 0.09], p = 0.180).

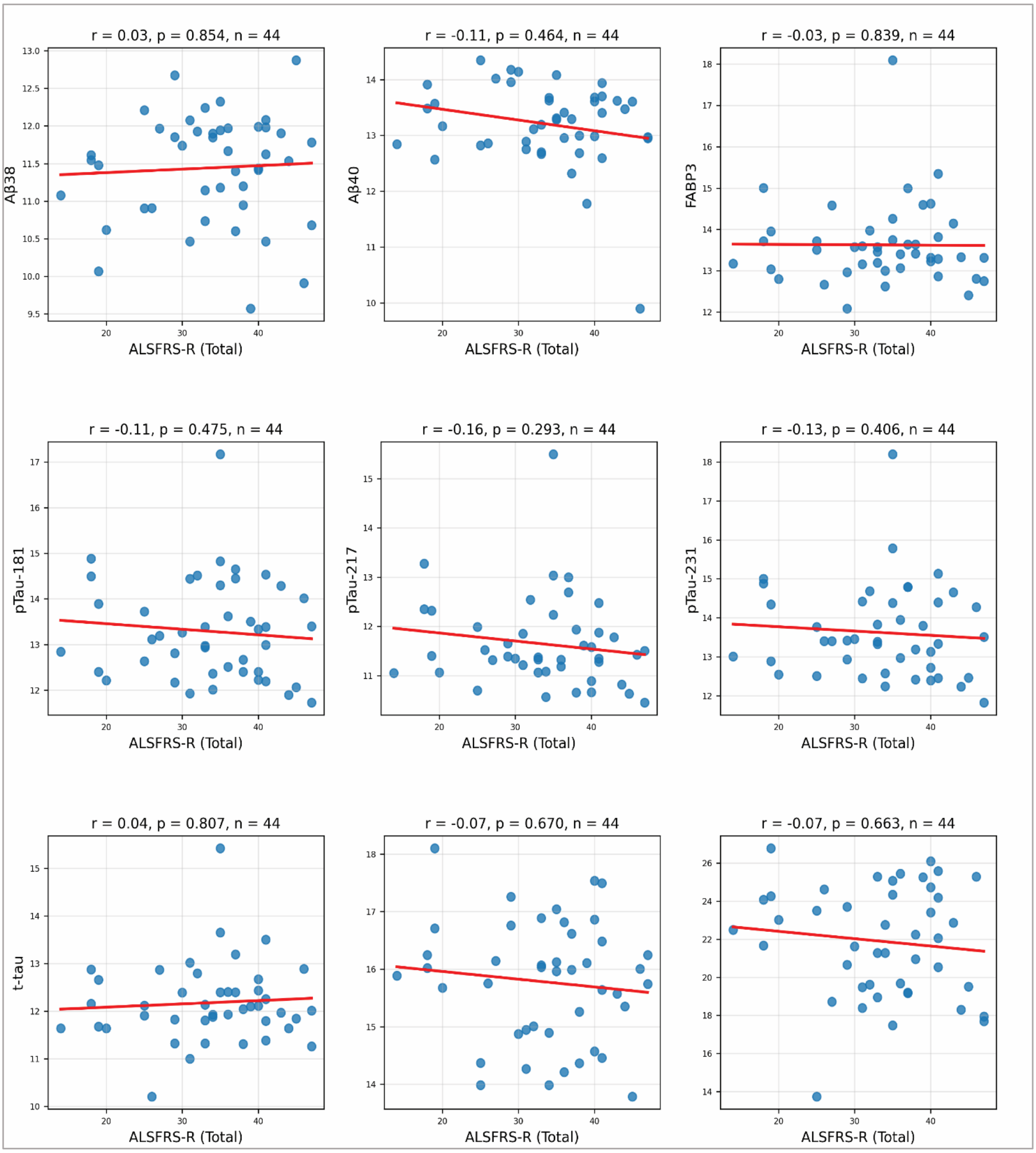

Supplementary Figure 7 – No significant correlation was observed between disease severity score and nine differentially expressed biomarkers in MND.

Serum samples from 48 MND patients were analysed using the NULISAseq™ CNS Disease Panel 120 kit. Samples with an IC median > 40% were excluded from further analysis (MND, n=1), resulting in a final sample size of 47 for statistical analysis. Of these, 44 individuals had an ALSFRS-R assessment recorded within 6 weeks of sampling. Each dot represents the result of a subject, with ALSFRS-R total score on x-axis and NULISAseq measurements (NPQ) on y-axis. Serum measurements showed no significant correlation between ALSFRS-R total score and NULISAseq markers for NfL (Spearman's Rank Coefficient R = -0.07, 95% CI = [-0.36, 0.24], p = 0.670), NfH (Spearman's Rank Coefficient R = -0.07, 95% CI = [-0.36, 0.23], p = 0.663),

Aβ38 (Spearman's Rank Coefficient R = 0.03, 95% CI = [-0.27, 0.32], p = 0.854), Aβ40 (Spearman's Rank Coefficient R = -0.11, 95% CI = [-0.40, 0.19], p = 0.464), FABP3 (Spearman's Rank Coefficient R =-0.03, 95% CI = [-0.33, 0.27], p = 0.839), pTau-181 (Spearman's Rank Coefficient R = -0.11, 95% CI = [-0.39, 0.19], p = 0.475), pTau-217 (Spearman's Rank Coefficient R = -0.16, 95% CI = [-0.44, 0.14], p = 0.293), pTau-231 (Spearman's Rank Coefficient R = -0.13, 95% CI = [-0.41, 0.18], p = 0.406), t-tau (Spearman's Rank Coefficient R = 0.04, 95% CI = [-0.26, 0.33], p = 0.807).

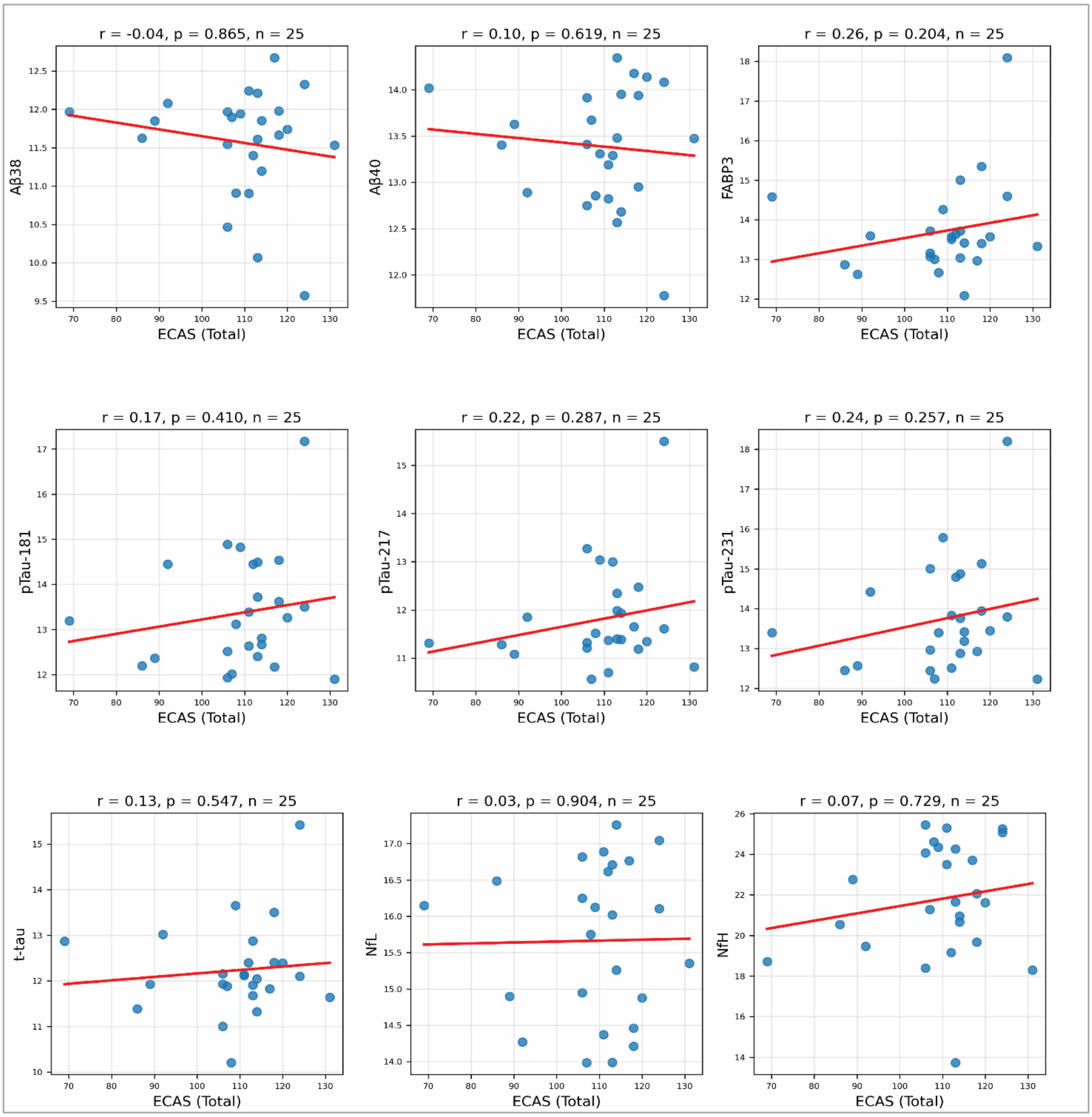

Supplementary Figure 8 – No significant correlation was observed between cognitive assessment score and nine differentially expressed biomarkers in MND.

Serum samples from 48 MND patients were analysed using the NULISAseq™ CNS Disease Panel 120 kit. Samples with an IC median > 40% were excluded from further analysis (MND, n=1), resulting in a final sample

size of 47 for statistical analysis. Of these, 25 individuals had an ECAS assessment recorded within 6 weeks of sampling. Each dot represents the result of a subject, with ECAS total score on x-axis and NULISAseq measurements (NPQ) on y-axis. Serum measurements showed no significant correlation between ECAS total score and NULISAseq markers for Aβ38 (Spearman's Rank Coefficient R = -0.04, 95% CI = [-0.42, 0.36], p = 0.865), Aβ40 (Spearman's Rank Coefficient R = 0.10, 95% CI = [-0.30, 0.48], p = 0.619), FABP3 (Spearman's Rank Coefficient R = 0.26, 95% CI = [-0.15, 0.60], p = 0.204), pTau-181 (Spearman's Rank Coefficient R = 0.17, 95% CI = [-0.24, 0.53], p = 0.410), pTau-217 (Spearman's Rank Coefficient R = 0.22, 95% CI = [-0.19, 0.57], p = 0.287), pTau-231 (Spearman's Rank Coefficient R = 0.24, 95% CI = [-0.18, 0.58], p = 0.257), t-tau (Spearman's Rank Coefficient R = 0.13, 95% CI = [-0.28, 0.50], p = 0.547), NfL (Spearman's Rank Coefficient R = 0.03, 95% CI = [-0.37, 0.42], p = 0.904), NfH (Spearman's Rank Coefficient R = 0.07, 95% CI = [-0.33, 0.45], p = 0.729).

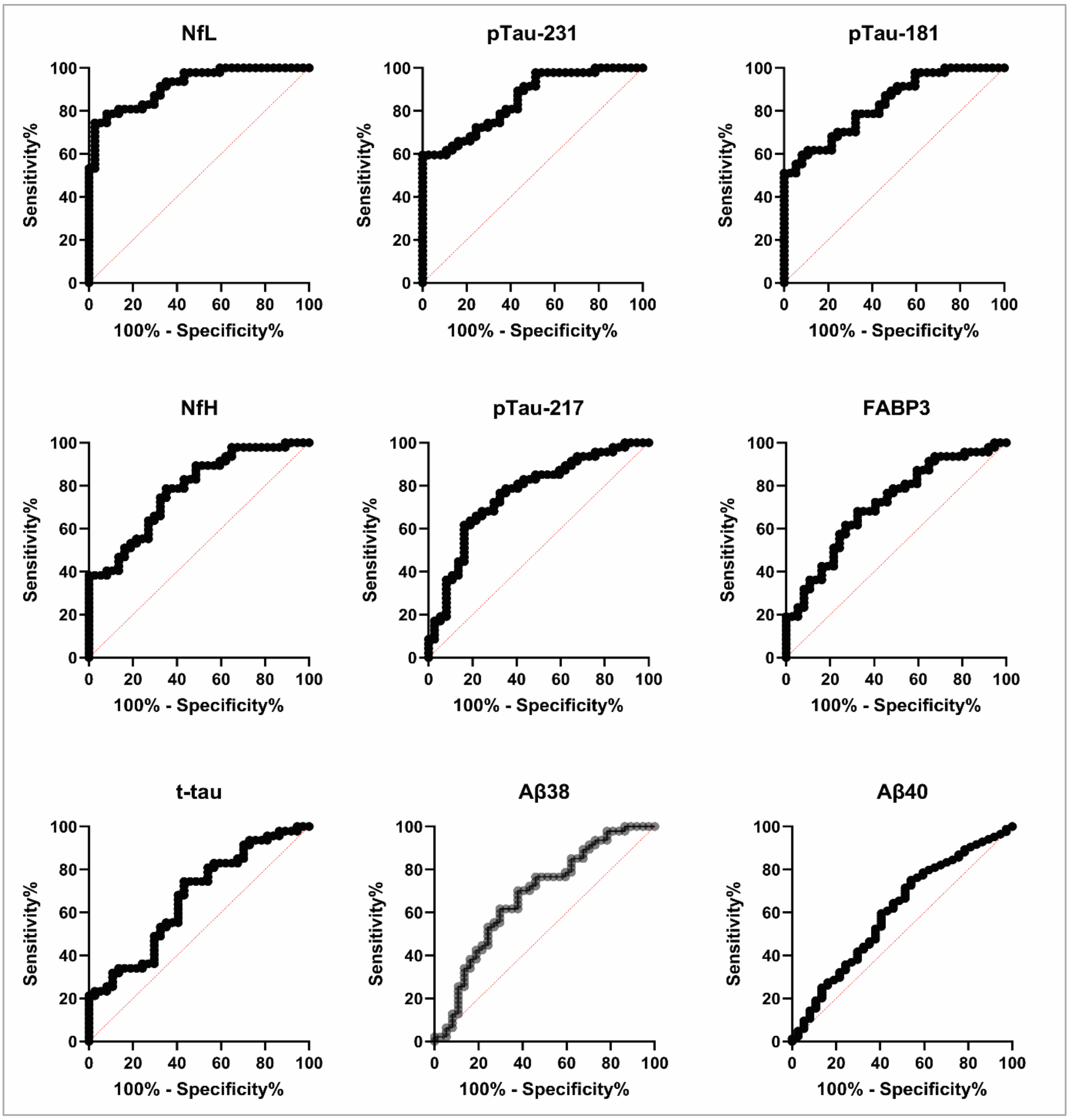

Supplementary Figure 9 – NfL, pTau-231, and pTau-181 showed high accuracy in differentiating MND cases from control. Serum samples of 86 individuals (MND, n = 48; non-ND control, n= 38) were analysed using a NULISAseq™ CNS Disease Panel 120 kit. Samples with IC median of > 40% were excluded from the analysis (MND, n= 1; non-ND, n = 1). This resulted in a final sample size of 84 for statistical analysis, comprising 47 MND and 37. A receiving operating characteristics (ROC) curves display sensitivity (%) and 100% - specificity (%) for NfL (AUC = 0.92, 95% Confidence Interval = [0.86 – 0.97]), pTau-231 (AUC = 0.85, 95% Confidence Interval = [0.77 – 0.93]), pTau-181 (AUC = 0.83, 95% Confidence Interval [0.75 – 0.92]), NfH (AUC = 0.78, 95% Confidence Interval = [0.68 – 0.88]), pTau-217 (AUC = 0.76, 95% Confidence Interval = [0.66 – 0.87]), FABP3 (AUC = 0.71, 95% Confidence Interval = [0.60 – 0.82), t-tau (AUC = 0.67, 95% Confidence Interval = [0.55 – 0.78]), Aβ38 (AUC = 0.68, 95% Confidence Interval = [0.56 – 0.80]), Aβ40 (AUC = 0.61, 95% Confidence Interval = [0.49 – 0.72]).

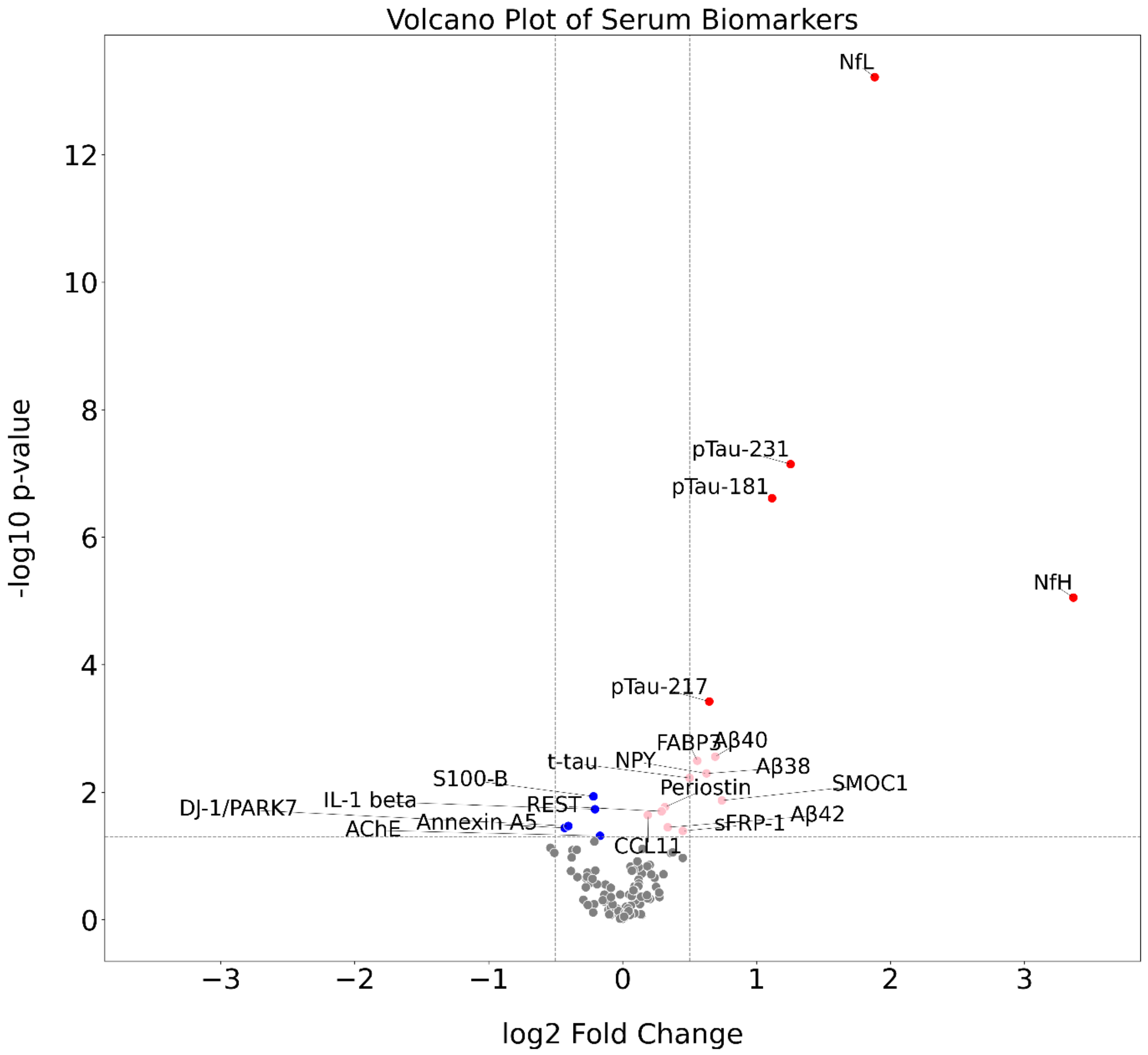

Supplementary Figure 10 – Serum proteins showed significant differences between individuals with ALS and control.

A volcano plot presents biomarkers that were significantly altered between individuals with ALS (n = 42) and non-ND controls (n = 37). The horizontal dashed line represents the unadjusted p value threshold of 0.05. Statistical significance was assessed using a two-tailed t-test, and p-values were adjusted for multiple testing using the Benjamini-Hochberg method. Grey coloured dots present proteins which did not show a significant difference between groups. Pink coloured dots highlight proteins that were significantly higher in ALS only when p value was not corrected (FABP3, log2FC = 0.56, P_{unadj} = 0.003; Aβ40, log2FC = 0.69, P_{unadj} = 0.003; Aβ38, log2FC = 0.63, P_{unadj} = 0.005; t-tau, log2FC = 0.50, P_{unadj} = 0.006; Aβ42, log2FC = 0.34, P_{unadj} = 0.035; NPY, log2FC = 0.63, P_{unadj} = 0.005; SMOC-1, log2FC = 0.74, P_{unadj} = 0.014; CCL11, log2FC = 0.19, P_{unadj} = 0.023; Periostin, log2FC = 0.32, P_{unadj} = 0.017; REST, log2FC = 0.29, P_{unadj} = 0.020; sFRP-1, log2FC = 0.45, P_{unadj} = 0.040). Blue coloured dots show proteins that were significantly lower in MND only when p value was not

corrected (AChE, log2FC = -0.17, P_{unadj} = 0.048; S100-B, log2FC = -0.22, P_{unadj} = 0.012; IL-1 beta, log2FC = -0.21, P_{unadj} = 0.019, DJ-1/PARK7, log2FC = -0.40, P_{unadj} = 0.034; Annexin A5, log2FC = -0.43, P_{unadj} = 0.036). Red coloured dots represent proteins that remained significantly higher in ALS after FDR correction (NfL, log2FC = 1.88, P_{adj} <0.001; NfH, log2FC = 3.37, P_{adj} <0.001; pTau-231, log2FC = 1.25, P_{adj} <0.001; pTau-181, log2FC = 1.12, P_{adj} <0.001; pTau-217, log2FC = 0.65, P_{adj} = 0.009).

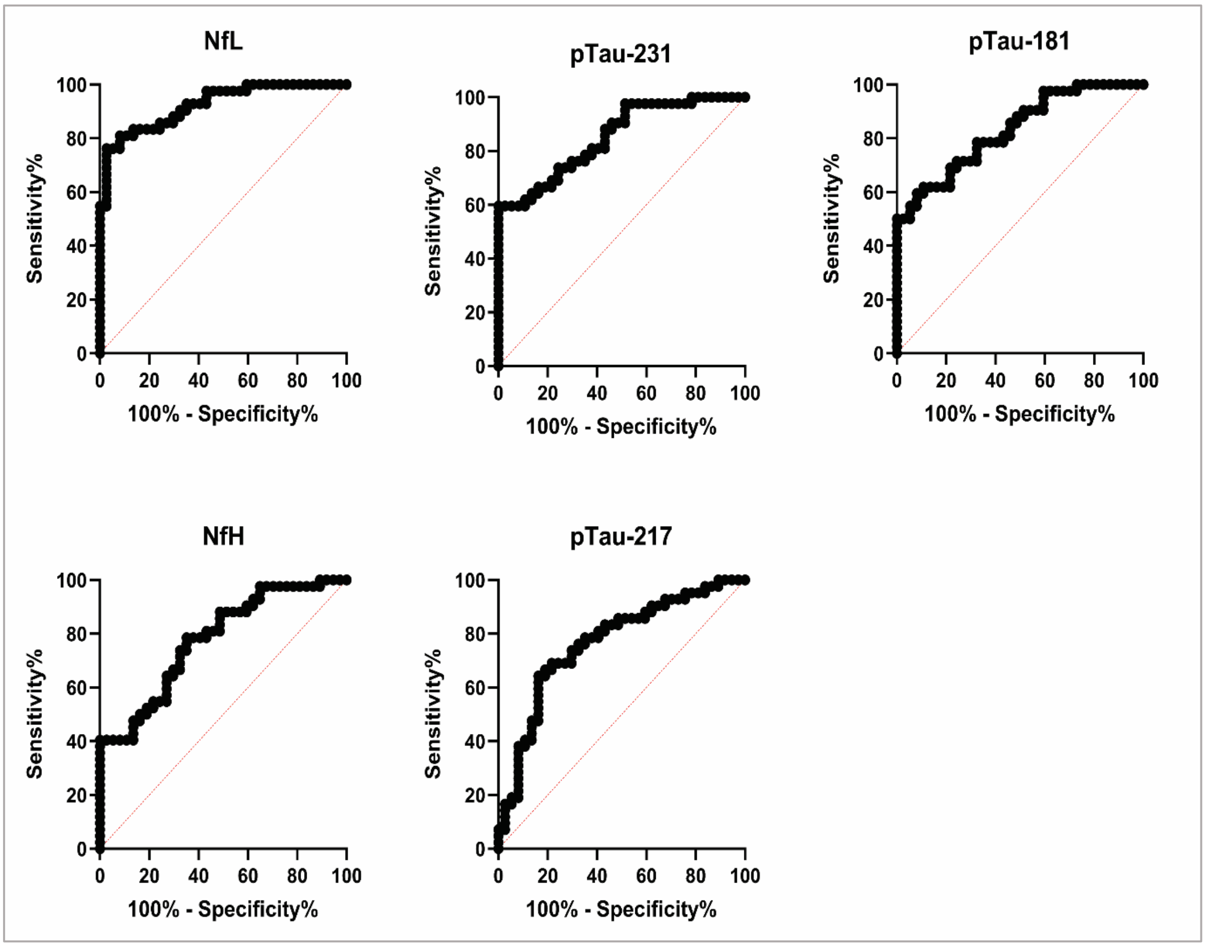

Supplementary Figure 11 – NfL, pTau-231, and pTau-181 showed high accuracy in differentiating ALS cases from control.

A receiving operating characteristics (ROC) curves display sensitivity (%) and 100% - specificity (%) for NfL (AUC = 0.92, 95% Confidence Interval = [0.87 – 0.98]), pTau-231 (AUC = 0.85, 95% Confidence Interval = [0.77 – 0.93]), pTau-181 (AUC = 0.83, 95% Confidence Interval [0.74 – 0.92]), NfH (AUC = 0.78, 95% Confidence Interval = [0.68 – 0.88]), pTau-217 (AUC = 0.77, 95% Confidence Interval = [0.66 – 0.87]) in differentiating ALS cases (n = 42) from non-ND control group (n = 37).

REFERENCES

1. Feng W, Beer JC, Hao Q, Ariyapala IS, Sahajan A, Komarov A, et al. NULISA: a proteomic liquid biopsy platform with attomolar sensitivity and high multiplexing. Nature Communications. 2023;14(1):7238.
